# Searching for Animal Sentience: A Systematic Review of the Scientific Literature

**DOI:** 10.3390/ani3030882

**Published:** 2013-09-04

**Authors:** Helen S. Proctor, Gemma Carder, Amelia R. Cornish

**Affiliations:** World Society for the Protection of Animals, 222 Grays Inn Rd., London, WC1X 8HB, UK; E-Mails: gemmacarder@wspa-international.org (G.C.); acornish@wspausa.org (A.R.C.)

**Keywords:** animal sentience, animal welfare, attitudes, behaviour, cognitive ethology, consciousness, emotions, subjective states

## Abstract

**Simple Summary:**

The emotional lives of animals is often doubted and questioned. Due to the subjective nature of animal emotions, many think that they are out of the reach of scientific measurement. In this systematic review, of over two decades of scientific literature, we found that this was not actually the case. By using a list of keywords, formed of both positive and negative emotions, and terminology relating to animal sentience, we reviewed the scientific literature. We found that the subjective lives of animals are not only a vital part of human medical research but are regularly measured and studied with scientific rigor.

**Abstract:**

Knowledge of animal sentience is fundamental to many disciplines and imperative to the animal welfare movement. In this review, we examined what is being explored and discussed, regarding animal sentience, within the scientific literature. Rather than attempting to extract meaning from the many complex and abstract definitions of animal sentience, we searched over two decades of scientific literature using a peer-reviewed list of 174 keywords. The list consisted of human emotions, terminology associated with animal sentience, and traits often thought to be indicative of subjective states. We discovered that very little was actually being explored, and instead there was already much agreement about what animals can feel. Why then is there so much scepticism surrounding the science of animal sentience? Sentience refers to the subjective states of animals, and so is often thought to be impossible to measure objectively. However, when we consider that much of the research found to accept and utilise animal sentience is performed for the development of human drugs and treatment, it appears that measuring sentience is, after all, not quite as impossible as was previously thought. In this paper, we explored what has been published on animal sentience in the scientific literature and where the gaps in research lie. We drew conclusions on the implications for animal welfare science and argued for the importance of addressing these gaps in our knowledge. We found that there is a need for more research on positive emotional states in animals, and that there is still much to learn about taxa such as invertebrates. Such information will not only be useful in supporting and initiating legislative amendments but will help to increase understanding, and potentially positive actions and attitudes towards animals.

## 1. Introduction

“Animals are like robots: they cannot reason or feel pain” (Descartes, 1596–1650). This quote may seem outdated when we consider both when it was said and what contradictory scientific evidence we have garnered since. However, when you consider that many non-human animals (hereafter referred to as animals) are treated inhumanely on a daily basis for the purpose of food, entertainment, research, and profit, the quote still seems relevant today. What stops us from taking the humane approach to agriculture, and what stops us from banning animal cruelty for entertainment? The arguments are often multi-faceted; disbelief or unawareness of animal suffering, lust for profit, or lack of empathy brought about by historical processes and layers of discourse around the moral value of animals [1]. Developing and sharing knowledge of animal sentience are key to addressing these arguments. Animal sentience refers to the ability of animals to feel and experience emotions such as joy, pleasure, pain and fear. It is animals’ capacity to feel both positive and negative states that drives the animal welfare movement and is the reason why animal protection laws exist [2,3,4]. 

Originally, concern for animals focused primarily on the animals’ physical health, with little thought for their mental well-being [5]. However, scientific interest in the subjective experience of animals has noticeably increased in the last 10 to 20 years (see [4] for a review). Animal sentience is sometimes dismissed due to the subjective nature of emotions and feelings; the building blocks of animal sentience, e.g., [6,7,8]. Whereas others argue that the complex and subjective nature of sentience should not be reason for its denial or dismissal as a robust science [4,9,10]. We feel that although sentience refers to subjective states it is not alone, as so does much of human psychology. The emotional experience of humans is both a personal experience and subject to false reporting [4]. We do not deny that humans are sentient because of this, but many do question animal sentience on the same basis. It appears therefore, that animal sentience is an unlucky victim of this scientific paradox. Whilst other areas of science will often make do with imperfect data, animal sentience is required to buck the trend and provide unequivocal proof [11,12]. Neuroscientist Donald Griffin coined the term “Paralytic perfectionism” to describe this contradictory way in which scientists still demand absolute certainty before they can accept animal sentience. He argued that the successful interpretation of mental states in others is a vital tool for social interactions, for both humans and animals [13]. 

Despite being subject to debate, descriptions of animal sentience, albeit in various forms, exist throughout the scientific literature. In fact, many experiments rely upon their animal subjects being sentient [14]. Analgesia studies for example, require animal models to feel pain, and animal models of schizophrenia are tested for a range of emotions such as fear and anxiety. Furthermore, there is a wealth of scientific studies, laws and policies which look to minimise suffering in the very animals whose sentience is so often questioned [15,16,17]. To overcome the paradoxical nature of the science of animal sentience, we sought to understand what is accepted and known about animal sentience in the scientific literature. The first challenge was to address the lack of consensus in regards to the definition of sentience. There is no universally accepted definition of sentience, and there are many different opinions as to where sentience exists in the animal kingdom [2,18]. We dealt with this by aiming to be as holistic as possible. The result was a peer-reviewed list of keywords comprised of primary and secondary emotions, technical terms, and traits commonly thought to be indicative of sentience. We were not intending to prove the strength or validity of these keywords in defining or proving animal sentience, but we instead wished to review what has been explored and discussed regarding the subjective states of animals. 

### 1.1. The Positive Side of Sentience

Although today, the subjective experiences of animals receive considerably more attention than 50 or even 20 years ago, research is still focused on the negative experiences of animals [19]. Whilst this research has been fundamental in improving many practices involving animals, it has failed to take into account the importance of positive experiences and emotions to the well-being of animals [20]. In more recent years, scientists have slowly begun to recognise that positive emotions and experiences are also a fundamental area of animal welfare science and key to ensuring a good state of animal welfare [5,20,21,22,23,24]. The emergence of new disciplines such as ‘Positive Psychology’ [25,26] and ‘Affective Neuroscience’ [27], which refers to both positive and negative effects, is evidence of this new focus. Progress continues to be slow however, and scientific understanding of negative emotions far outweighs that of positive emotions, both in animals and humans [20]. In this study we aimed to review what is assumed and explored in the scientific literature in regards to the positive and negative aspects of animal sentience and the impact this has on animal welfare. 

### 1.2. Mammalcentrism

Animal sentience research is often accused of being mammal-centric. This is primarily due to the similarity of physiology and neurology in humans and other mammals, and the relative ease of drawing conclusions from argument-by-analogy [12,28]. In addition, attitudes to animals may be affected by innate human tendencies to sympathise with animals depending on their status, use, attractiveness, or believed intelligence [29,30]. Yue-Cottee for example, describes how cold-bloodedness is often used as a reason for the denial of subjective feelings to fish. She argues that a metabolic difference should not be used as a reason for denying them concern or protection, particularly in light of the contradictory scientific evidence [12]. There is hope, however, and science is slowly moving away from this dominant, mammalcentric perspective. For instance, in recent years we have seen a growing focus on the subjective minds of invertebrates such as cephalopods and decapod crustaceans [31,32,33]. As the field of animal sentience research continues to grow, scientists should be able to further develop the methodologies used to explore the affective states of animals. The resulting increase in scientific knowledge on the abilities of animals will hopefully help to change people’s perceptions of animals and will have varying implications for practices and industries. In this study we have reviewed articles published from 1990 to 2012 and identified the taxa being studied. This has allowed us to evaluate the progression of research and knowledge of animal sentience, understand what is known about the different taxa, and to identify the remaining gaps in our knowledge. 

### 1.3. Humane Research

Whether a study design impairs the welfare of the animal subjects remains one of the greatest ethical paradoxes of animal sentience research. Although many studies using animals will have been subjected to some level of ethical review, this does not necessarily mean the study has not significantly impaired the welfare of the animals involved. For example, methodologies involving inhumane procedures can be approved due to the potential of the results to justify the suffering [34]. Furthermore, a lot of un-moderated animal research still continues around the world [35,36]. It is likely that this situation will improve as the focus of animal sentience and welfare research shifts on to the study of positive emotional states. The objective of such studies would encourage the promotion and evaluation of positive emotions, rather than negative ones. 

When research must involve animals, one possible change is to address how the animals are housed for these studies. The issue of housing has received a lot of attention in terms of enriched cages and naturalistic settings [37,38], but there is even greater scope for improvement when you address the issue of housing and breeding as a whole. For instance, when research aims to explore animal behaviour for greater ethological understanding, there are many alternatives to laboratories that should at least be explored. For example, existing populations of pet animals or animals in shelters, zoos, farms, or in the wild can often provide the subjects required for research. In fact, such populations can provide a more realistic model of the species than a laboratory bred animal [39]. In this study we documented where the animals were housed or where the studies took place, for example, were they zoo or laboratory animals? We also recorded the main purpose of the study, for instance, did the study seek to develop knowledge of animal behaviour or improve animal welfare? We then examined the relationship between these data to understand how the animals were housed for each of the main purposes and we drew conclusions regarding the potential for welfare improvements.

### 1.4. The Importance of Animal Sentience Research

Understanding animal sentience has many benefits to humans, animals and science. Too much scepticism, particularly when unfounded, hinders scientific process and positive change for animals [40,41]. Furthermore, accepting the existence of affective states in animals can be an important step towards tackling other key problems in neuroscience [14]. The many parallels between the subjective experiences of animals and humans are clearly utilised in research that requires animal models for human afflictions [27]. Most importantly, knowledge of what animals experience, what is important to them, and what constitutes a good life for them, is key to truly improving their welfare. Just like for humans the experience of positive emotions, such as joy and pleasure, has meaningful bearings on the mental and physical welfare of animals [16,42].

We sought to address the lack of consensus on the prevalence of animal sentience by extensively reviewing the scientific literature. We analysed the progression of published research discussing and exploring various aspects of animal sentience over a focal period of 22 years. The results highlight what is being explored and what is already assumed in regards to animal sentience, and in which taxa. As the human population continues to grow so does the number of animals we use for our own means. Understanding the subjective minds of animals is therefore of utmost importance to their welfare. We hope that the findings of this paper can highlight where future research is needed in the field of animal sentience and the importance of what we already know. 

## 2. Materials and Methods

### 2.1. Keywords

We compiled a list of emotions, traits, and terminology associated with or indicative of animal sentience using three existing lists of human emotions [43,44,45], and 22 keywords specific to animals and animal sentience (Appendix Table A1). These words were derived from literature reviews performed prior to the start of the study. Each keyword was extensively defined to ensure only reference to the subjective experiences of animals was considered in the review. The final list of 174 keywords was then peer-reviewed and approved by a scientist in the field of animal sentience [46]. 

### 2.2. Literature Search

We searched two journal databases; Science Direct and Ingenta Connect, for articles from peer reviewed journals, indexed since 1990, containing both the keyword, and the word ‘animal’ in the abstract, title or keywords. The focal period of 1990 to 2012 was chosen because it allowed for a large and recent study period, yet it was still feasible given our time restraints. We then filtered the results according to the following criteria. Firstly, we removed any books, short communications, letters, non-English articles, review papers, and articles without abstracts, leaving only original, full research articles. Secondly, we removed any articles that were not using animals but were only referring to previous studies or findings from animal research. Finally, we only retained articles that used the keyword in line with the detailed definition and in reference to the animals’ subjective state. For example, stress as an emotional state was recorded, whereas reference to stress as a physiological state, such as heat stress, was omitted. 

Each of the authors took part in collecting the data, and so to ensure consistency, each keyword and category used in the study was fully defined with working examples to reduce the degree of subjectivity. Furthermore, inter-observer reliability tests were performed for each aspect of the data collection (e.g., article selection and categorisation) throughout the study period. Reliability exceeded 95% agreement upon each of the tests. 

### 2.3. Research Questions

After the initial sorting phase, we answered a number of questions for each article abstract. To start with we looked at whether the study assumed or explored the existence of the keyword in the animal subjects. For example, a study could explore whether rats can experience pain, or it could measure the pain experienced by rats following analgesia. The latter accepts that rats can feel pain and uses that knowledge, whereas the former is exploring whether or not rats can experience pain at all. Both types of study were reviewed, in order to measure the acceptance of animal emotions in the scientific literature and to establish which aspects of animal sentience have been experimentally explored. 

To determine the number of articles referring to positive and negative keywords, we labelled each of the keywords as positive, negative or neutral, depending on the valence of the emotion or trait depicted. For example, the keyword pain was labelled as negative, whereas the keyword pleasure was positive. For the neutral keywords the valence was defined at the individual article level wherever appropriate. For example, the use of the term ‘affective state’ in a study could have referred to either a negative or positive affective state, or both, whereas the keyword ’theory of mind’ had no valence and remained neutral. 

We then asked which year the article was published. When analysing this question we only looked at the data returned from the years 1990 to 2011. This was because the 2012 results were not representative of the entire year due to the timings of the data collection, which took place in mid-2012. All of the other questions looked at the entire 1990–2012 period. 

To determine whether any observed differences were unique to the articles reviewed or merely reflective of the general trends in publication numbers, we looked at the total number of articles published in Ingenta Connect and Science Direct in the years 1990 and 2011. For consistency we used the same search criteria as before but without the keyword. For example, an advanced search was performed in both databases to determine the total number of papers published in 1990 with the word ‘animal’ in the title, abstract or keywords. We then determined the percentage increase or decrease between these years for both the total number of papers published and for our reviewed papers. 

The remaining questions probed for further details of the animals used in the study. We looked at which taxa were being studied, recording the sub-phylum, order, class and species or common name of the animals used in each study. When possible we identified the experimental setting of the study from the article abstract. For example, did the research take place in a laboratory, a zoo, or on a farm? Research farms were labelled as ‘farms’, due to the similarity in the housing environment for the animals. Finally, we determined what the primary purpose of the study was, recording whether the research was performed for human benefit, such as a pharmaceutical study, to advance knowledge of animal behaviour, to further knowledge of animal sentience, or to improve animal welfare.

### 2.4. Data Analysis

We organised the data into two spreadsheets; version one (V1) was the original intact spreadsheet, and version two (V2) had the duplicate articles removed (some articles referred to more than one keyword). We used V1 for the analyses that looked at individual keywords, such as the number of articles returned for each keyword. Finally, we used V2 for the analyses that required us to look at the data set as a whole without the duplicate entries. For example, the number of articles published in 1990 *vs.* 2011.

The primary analysis was descriptive to allow us to review the relationships between the different research questions and to identify appropriate sample sizes for statistical analysis. Following this we used the chi-square goodness of fit test to identify significant differences between the number of assumed and explored articles, the numbers recorded for each sub-phylum, the purpose of the studies, the experimental setting, and the numbers of articles published in 1990 compared to 2011. All analyses were performed using Statistical Package for the Social Sciences (SPSS) version 21 for Windows. Statistical significance was indicated by *P* < 0.05.

## 3. Results

We collected a total of 2,804 papers from all of the searches performed; dropping to 2,562 once the duplicate entries were removed. Forty-three keywords out of the total 174 returned suitable results, ranging in number from one to 635 articles per keyword. From these keywords, eight were labelled positive, 23 were negative, and 12 were either neutral or dependent upon the individual article.

### 3.1. Why?

Animal sentience was not the primary reason for why any of the studies were performed, and it was only deemed to be a secondary or subsequent purpose for five of the articles we reviewed. Instead, we found there to be three over-arching reasons for the studies, and these were; human benefit, animal welfare and animal behaviour. Significantly more studies were performed for human benefit (e.g., pharmaceutical development), than there were for either animal welfare or animal behaviour reasons (*X^2^* = 1,462.34, *df* = 2, *P* < 0.001). There were also significantly more studies performed for animal welfare reasons than there were for animal behaviour reasons (*X^2^* = 9.94, *df* = 1, *P* < 0.05). 

### 3.2. Who?

**Figure 1:**
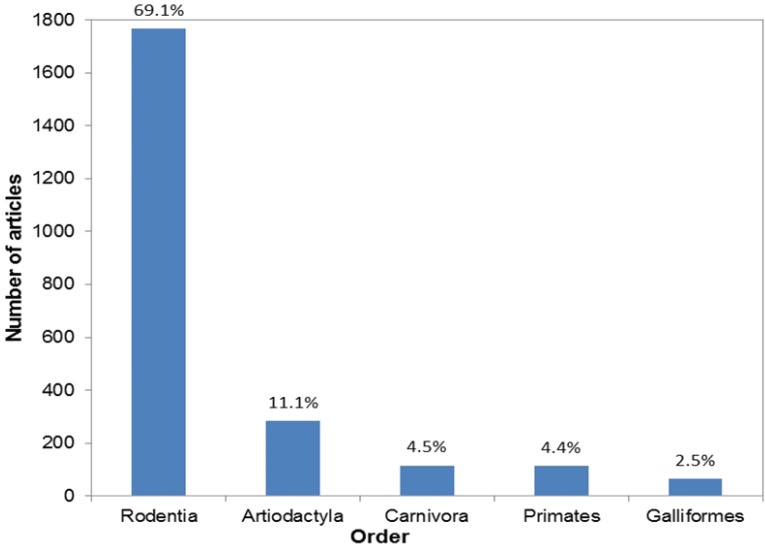
The number of reviewed articles using each of the top five orders. Data labels refer to the percentage of the total articles.

**Figure 2:**
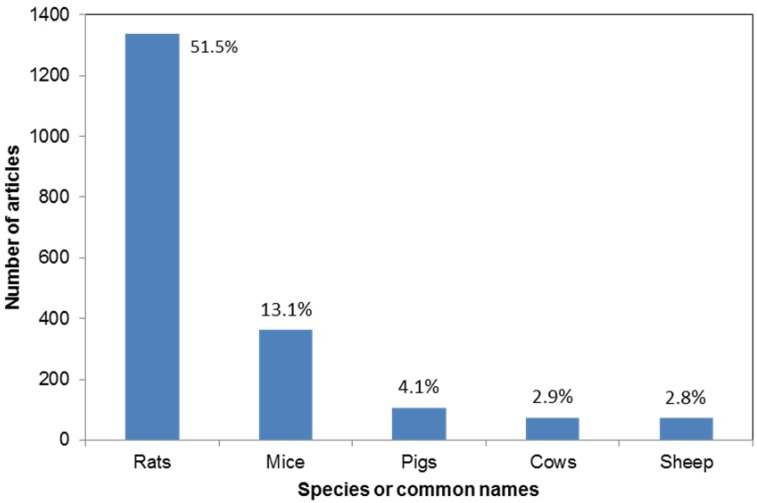
The number of reviewed articles using each of the top five species or common names. Data labels refer to the percentage of the total articles.

We captured detailed information about the animals used for each article, and found that, overall, vertebrates (n = 2,519) were used significantly more than invertebrates (n = 32, *X^2^* = 2,424.61, *df* = 1, *P* < 0.001). These two sub-phyla were comprised of 12 taxonomical classes; six vertebrate and six invertebrate. Mammalia was the most popular class of animals used (n = 2,346, 91.89%), followed by Aves (n = 116, 4.54%), and Actinopterygii (n = 45, 1.76%). Climbing down the taxonomical tree we found that these classes gave way to 57 orders, 11 of which were invertebrates, and the remaining 46 were vertebrates. The top five orders and species are shown in Figure 1, Figure 2.

Because the human benefit studies comprised the majority of the articles we reviewed (n = 1,765), we also looked at the results with those articles removed to see whether there were any differences in the returned results. We found no differences in the use of vertebrates and invertebrates, with the majority of studies still using vertebrates (vertebrates: n = 766, 96.47%, invertebrates: n = 28, 3.66%). Mammalia, Aves, and Actinopterygii were still the most popular classes used (Mammalia: n = 610, 76.73%, Aves: n = 110, 13.84%, Actinopterygii: n = 36, 4.53%). However, there was a difference for the orders; Rodentia, which were used for 69.07% of the articles overall, were only used for 9.91% of the articles once the human benefit studies were removed. The top five orders changed to Artiodactyla (n = 277, 35.24%), Carnivora (n = 110, 13.99%), Primates (n = 90, 11.45%), Rodentia (n = 79, 9.91%), and Galliformes (n = 60, 7.53%). The top five species changed to pigs (n = 100, 12.55%), cows (n = 73, 9.16%), sheep (n = 67, 8.41%), chickens (n = 48, 6.02%) and rats (n = 48, 6.02%).

### 3.3. Assumed or Explored?

Of the 2,562 articles we reviewed, 2,546 of them referred to a keyword as assumed; an accepted trait or emotion already deemed to be present in the animal subjects. A further 16 of the articles explored whether or not the animals experienced the trait or emotion. There were significantly more articles assuming the keywords (n = 2,546) than there were studies exploring their existence (n = 16) (*X^2^* = 2,497.4, *df* = 1, *P* < 0.001). Looking more closely we found that the vertebrate bias was apparent in both the explored and assumed studies. Out of the 16 explored articles only two were studying invertebrates and only 29 of the 2534 assumed articles looked at invertebrates. 

### 3.4. Keywords

We found that 74% of the articles arose from just five keywords. These were fear (n = 636, 22.68%), stress, (n = 607, 21.65%), pain (n = 305, 10.88%), anxiety (n = 267, 9.52%), and depression (n = 222, 7.92%). These words also posed data collection difficulties. Each of these keywords returned between 1,409 to 2,026 results from the initial Science Direct search and unfortunately Science Direct only allows you to view the first 1,000 returned articles. These searches were therefore clipped at 1,000 articles, compared with the other keywords that returned less than 1,000 articles. Had the data been collected from the full list of returned articles these keywords would still remain the top five. It is expected however, that there would have been a higher number of returned articles for each of these keywords, and they would not necessarily remain in the same order. When we removed the human benefit studies from the analysis we found that the top keywords differed. The top five keywords changed to stress (n = 223, 27.98%), fear (n = 142, 17.82%), aggressiveness (n = 139, 17.44%), play (n = 60, 7.53%), and distress (n = 42, 5.27%). It is possible that the sampling issue may have also affected these figures. 

Some of the keywords with returned results were assumed in a range of species and orders. For example, the keyword ‘aggressiveness’, which referred to the emotional state, rather than simply aggressive behaviour, was assumed in 34 out of 57 orders. Seven of these were invertebrate orders, which meant that ‘aggressiveness’ was assumed in 63.64% of the invertebrate orders recorded in the review. The keyword ‘stress’, which referred to emotional stress, was assumed for 31 different orders, 29 of which were vertebrates and two were invertebrates. ‘Fear’ was an assumed emotion for 17 of the orders, one invertebrate and 16 vertebrates. None of the keywords were both explored and assumed for the same species or order, within a two year period of publication. 

### 3.5. Positive or Negative?

There appears to be a greater tendency for studies to assume the existence of negative states in animals than positive ones. Out of the 2,546 ‘assumed’ articles, only 154 of them referred to positive states or experiences in animals, compared to 2,359 articles which referred to negative keywords. The remaining 31 articles were classed as neutral and discussed keywords that had no valence, such as theory of mind or consciousness. In the ‘exploring’ studies we found the opposite to be the case, with 11 out of 16 articles looking at positive keywords, compared to just five articles looking at negative ones, however the sample size was too small for any analysis. When we removed the human benefit articles from both the explored and assumed studies we found the negative bias was still present. There were only 149 articles referring to positive states that were performed for animal welfare or behaviour reasons, compared to 625 articles referring to negative states. Furthermore, studies looking at positive emotions and keywords were more likely to be performed to develop knowledge of animal behaviour (n = 99, 29.29%), compared to animal welfare (n = 49, 11.32%), or human benefit reasons (n = 15, 0.85%). 

### 3.6. Where?

We noted 10 different types of experimental or observational settings in the review. From these, laboratories were used the most (n = 2,018, 78.92%), followed by farms (n = 323, 12.63%), the wild (n = 109, 4.26%), zoos (n = 43, 1.68%), and pet households (n = 33, 1.29%). The remaining five categories ranged in number from one to 20 articles and comprised of stables, circuses, shelters, sanctuaries, and stray animals (domestic). Laboratories were clearly used the most, but both laboratories and farms were recorded significantly more than the other eight categories (*X^2^* = 2,497.4, *df* = 1; *P* < 0.001). When we removed the human benefit studies we found similar results, although laboratories were less likely to be used for these studies (farm: n = 320, 40.40%, laboratory: n = 257, 32.45%, wild: n = 13.13%, zoo: n = 40, 5.05%, and pets: n = 28, 3.54%). When we looked at what type of keywords were being studied, we found that pet and zoo animals were more likely to be studied for positive keywords (pets: n = 12, 35.29%, zoo: n = 15, 34.88%) than laboratory (n = 73, 3.64%), farm (n = 41, 13.36%), or wild animals (n = 20, 18.02%). 

### 3.7. When?

**Figure 3:**
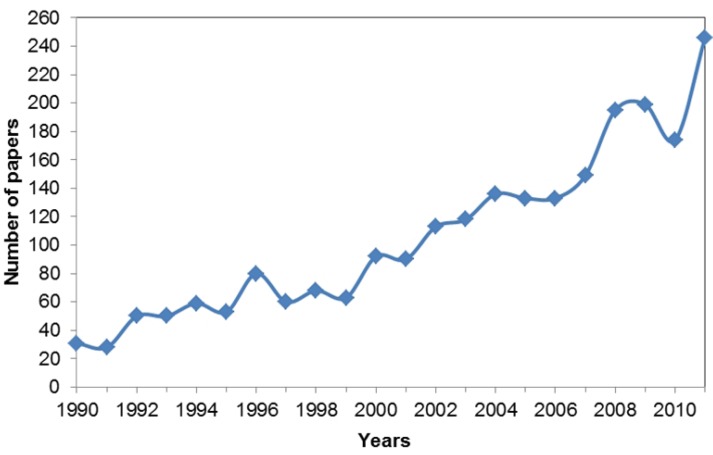
The total number of reviewed articles published from 1990 to 2011. The 242 articles published in 2012 were not included in this analysis as the data collection period did not account for the entire year.

**Figure 4:**
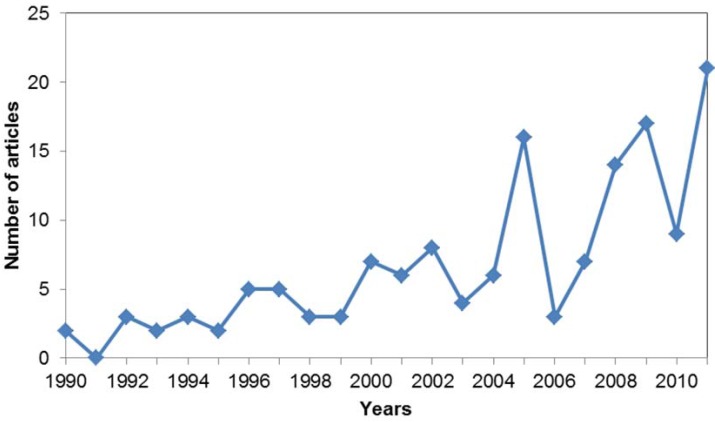
The number of reviewed articles containing positive keywords, published between 1990 and 2011.

**Figure 5:**
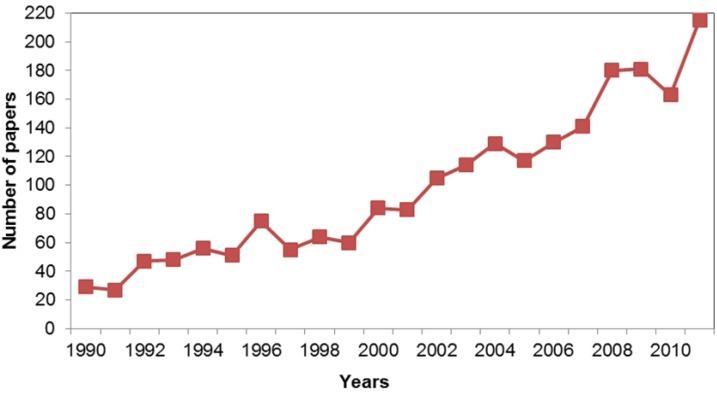
The number of reviewed articles containing negative keywords, published between 1990 and 2011.

The number of published articles discussing the sentience-related keywords has increased over the past two decades (Figure 3). We compared the number of articles published in 1990 and 2011 and found there were significantly more articles published in 2011 than in 1990 (*X^2^* = 166.88, *df* = 1, *P* < 0.001). This represented a 693.54% increase in articles published in 2011 compared to 1990. In comparison, there was a 249.25% increase in the number of articles published in Science Direct and Ingenta Connect in 2011 compared to 1990, with the word ‘animal’ in the abstract, title or keywords. The increase in publications is also consistent for both the positive (Figure 4) and negative articles (Figure 5). There were significantly more articles published in the year 2011 compared to 1990, for both the positive (*X^2^* = 15.7, *df* = 1, *P* < 0.001) and negative studies (*X^2^* = 141.788, *df* = 1, *P* < 0.001). Studies being performed for each of the three ‘why’ categories also significantly increased from 1990 to 2011 (animal behaviour; *X^2^* = 33.62, *df* = 1; *P* < 0.001; animal welfare; *X^2^* = 30.19, *df* = 1, *P* < 0.001; and human benefit; *X^2^* = 104.26, *df* = 1, *P* < 0.001).

## 4. Discussion

Animal sentience is often thought of as a complex, poorly defined, subjective, and abstract concept, raising as many questions as it does answers. If you talk to different people about their views on animal sentience you will undoubtedly get various responses. If you asked a pet owner whether animals have feelings, they would regale you with stories of when their dog comforts them, or is proud of himself when he opens a closed door. A farmer who works closely with his or her animals may tell you about how cow number 19 likes being tickled behind her ears, and how cow number 25 is the shy and cautious one. But then you may talk to someone who sees animals only in terms of their monetary value. Animals to such people are not living, sentient beings, they represent commodities. It is far easier for them to see animals in this way but far less convenient for them to consider their ability to suffer or their need to experience positive emotions like pleasure. How people perceive animals is never black and white, attitudes may depend on the species in question, and the animals perceived mental ability [47]. However, when you consider how we treat the animals we farm for food, experiment upon, or use for entertainment, you can clearly see how important these perspectives are, and which the dominant ones are. 

### 4.1. Why?

Although our review recorded the use of a wide range of species and keywords, the majority of articles referred to the top five keywords; fear, stress, pain, anxiety, and depression, were performed for human benefit, and used rats and mice. Such a result is unsurprising when you consider the dependence of research upon rodents, and that their ability to feel and experience emotions is often both beneficial and essential to animal model research. This is interesting however, when you consider that much of the criticism around animal sentience science is concerned with the inability to measure subjective states [6,7,8]. In the 1,765 studies performed for human benefit, the subjective states of animals were not only measured but were often fundamental to the research being performed. The primary aim of these studies may not have been to measure animal sentience, but the scientists responsible have, perhaps unwittingly, assumed and measured its existence for the purposes of their studies. Given that this type of research is often looking at the development of drugs for human use, the scientific standards for this research should be of a very high standard and subject to extensive scrutiny. It could therefore be safely assumed that their measures of subjective states in animals are not leaps of faith but are instead based upon robust, empirical data. If this is the case, then it would provide strong evidence for the credible and objective nature of animal sentience research, and offer powerful rebuttals to criticisms which maintain the opposite to be true. 

### 4.2. Who?

We can see from the results that industry and human medical progress are major factors influencing which species are studied. The pharmaceutical industry relies heavily upon rodents to act as animal models for human disorders, such as depression and anxiety [48,49]. Moreover, the billions of animals used in agriculture every year further outweighs the recorded numbers of research animals [36,50]. It is therefore unsurprising that rats, mice, pigs, cows, sheep and chickens were the top species used in the studies reviewed. However, very few of the studies looked at fish. Fish are increasingly being farmed and billions are wild-caught every year [51], and they are increasingly being used in experimentation [52]. As a result, we would have expected fish to feature more frequently than the 45 times we recorded in the review. The shortage of research on fish may be a result of the lack of consensus around fish sentience. Despite fish often being protected in legislation and in research regulations, some still argue that they are incapable of feeling pain [6,8]. In recent years, several studies have suggested that fish do have the capacity to feel pain, despite claims that their neurology renders them incapable of such experience [8,53,54,55]. We hope therefore, that future reviews will feature fish more frequently as a result of the growing understanding of their subjective states.

Invertebrates are used and managed on a considerable scale. They are killed during pest control, experimented upon, and both consumed and farmed on an increasing scale every year [56]. In addition, human reliance on invertebrates is expected to intensify, as they are increasingly being viewed as a viable and sustainable food source for the growing human population [57,58]. Considering the increased impact we have on invertebrates, and the fact that invertebrate species comprise 99% of the world’s animals [59], we were disappointed to see how little they featured within the scientific literature. The treatment of invertebrates differs greatly to that of vertebrates, due to the difference in attitudes towards these animals, and the lack of understanding about their capacity for subjective feelings [28,59]. In recent years, as a result of increased understanding of the subjective states of invertebrates, several positive developments regarding their protection have come about. For example, the UK’s Animals (Scientific Procedures) Act (1986) was updated in 2012 to include all cephalopods and New Zealand’s Animal Welfare Act (1999) includes both crabs and crayfish. Research into the subjective states of invertebrates must continue to ensure that all sentient invertebrate species are protected. 

### 4.3. Assumed or Explored?

We performed this review to scratch beneath the surface of animal research, to understand what is being explored, and in whom. What we found surprised us; very little is actually being explored. A lot of these traits and emotions are in fact already being accepted and utilised in the scientific literature. Indeed, 99.34% of the studies we recorded assumed these sentience related keywords in a number of species. In comparison, there were only 16 studies exploring the existence of these traits in animals, and these took place across the entire study focal period and were not seen to increase in recent years. The small number of studies exploring the capacity of emotions in animals suggests that such explorative studies are not increasing, as has previously been suggested, e.g., [12,20]. In view of the importance of animal sentience research to the welfare of animals, we hope that we will see an increase in the future, as more scientists continue to explore animal sentience. 

### 4.4. Positive or Negative?

Each of the top five keywords were negative, and there were far fewer articles discussing the positive keywords than the negative ones. Each of the top keywords referred to states in animals that are intrinsic and necessary for fitness and survival, but extended experience of them can be detrimental to their welfare. The large number of studies discussing the negative keywords is still a positive outcome though, as good animal welfare is dependent upon the absence of these. It is however, increasingly being recognised that good animal welfare also requires the promotion of positive states such as pleasure [5,23,24]. By looking at each ‘why’ category separately we can present some possible explanations for the overwhelming bias for negative states. The human benefit studies in this review were mostly performing research into human physical and mental health. Human research has the same bias for focusing on negative emotions as animal sentience research does [20]. It is therefore unsurprising that the majority of animal research performed for human benefit has the same negative bias. For the animal welfare and behavioural studies the lack of discussion and exploration of positive emotions is a greater concern and we had hoped for a more balanced focus. The bias in these sectors may be reflective of the historical focus on negative states and the relative recent shift in attention towards the promotion of positive states in animals. The discussion of positive keywords did increase over the 21 year focal period, and comparisons between 1990 and 2011 showed a significant increase. This is an encouraging result and shows that reference to positive states is increasing. However, Figure 2 shows that it has not been a steady or consistent increase. These results were disappointing but not unanticipated, as the bias towards negative states in animals has been discussed before [4,20,23,60]. There are associated difficulties with measuring positive states in animals, which may give further indication as to why the focus is so biased towards negative states. For example, emotions such as fear and pain are often far more intensely communicated and expressed than positive emotions, making them easier to identify [22,53,54]. This in turn often creates a sense of importance and urgency to the issue. Fortunately there is success from those scientists seeking to tackle these issues, and new approaches for measuring positive emotions are appearing, e.g., [16,61,62,63]. 

Animal welfare science needs to move away from the bias towards negative states. Although addressing negative states is a fundamental step in addressing animal welfare, failing to recognise the importance of positive experiences and emotions can have detrimental effects on both the science of animal welfare and the well-being of the animals we use. When we focus on negative states we are only addressing half of the problem. Animals have an interest in positive experiences in the same way humans do, and so positive experiences and emotions warrant much more consideration than they currently receive [4]. This one-sided approach to welfare over-simplifies the motivations and needs of animals [5] and fails to recognise some of the benefits that positive emotions may have on the animals’ mental and physical health. For example, in humans it is thought that humour and laughter may benefit health, and humour is increasingly being incorporated into human medical care [20,64]. Furthermore, a more holistic knowledge of animals’ emotional state may be helpful in predicting the responses of animals to certain situations [20]. Knowledge such as this would have significant practical applications to many situations where animal welfare needs to be improved. 

### 4.5. Where?

Of the 10 experimental settings recorded, laboratories were used the most, coming only second to farms once the human benefit studies were removed. This is unsurprising considering the number of human benefit studies performed where laboratory settings are the standard. The animal behaviour category consisted of 106 laboratory studies out of a possible 350. This was surprising given that these studies were performed primarily to further ethological knowledge. We do not wish to criticise such research or question its value, after all, knowledge of animal behaviour is integral to understanding animal sentience. We also acknowledge that laboratories offer the standardised settings that are sometimes required for such studies. We would however, like to highlight that there are also a number of other suitable settings where research can be performed. Moreover, on many occasions these can provide a truer representation of the species behaviour than an artificial laboratory setting can. Breeding animals for a laboratory existence should always be seriously considered given the welfare implications of laboratory research and housing. Wherever possible, existing populations such as farm, wild, zoo, or pet animals should be utilised to avoid the unnecessary over-breeding and discomfort laboratories often inflict [65]. 

### 4.6. When?

Overall, the number of published articles reviewed had increased from 1990 to 2011 (Figure 1). When we compared the percentage increase of the reviewed papers to that of the total number of papers published, we found that the increase was far greater for the studies reviewed (693.54% *vs.* 249.25%). This suggests that the observed increase in papers referring to the keywords can be attributed to a specific increase in the use of these sentience related keywords and not attributable to a general increase in publication. This is a positive result, and we hope that as acknowledgment of animal sentience increases, this will in turn have a positive impact on how we view and treat animals.

### 4.7. Limitations and Future Research

Our results have provided a beneficial and original insight into the issue, but because we only looked at two journal databases they are not inclusive of the entire body of scientific literature. Future work would therefore benefit from incorporating other databases and also the non-English literature, which was excluded in this study. Our results provide information about articles published in 1990 through to mid-2012, and we would like to continue to review future research on a bi-annual basis.

Due to time and budgetary constraints we were only able to review the abstracts of the articles and not the full papers. Although we could identify the information we required for most of the time via this method, there were a few instances when we could not determine which species were being used. Furthermore, the keyword searches performed in the journal databases only searched the abstract, title and keywords. As a result, some articles which only used the keyword or the word ‘animal’ in the main text and not the abstract, title or keywords, would have been excluded from the review. Sample searches performed in the pilot phase of the study showed minimal differences in the number of valid articles returned from this method, *vs.* searches performed using the entire article. Future work could look at analysing the entire papers to confirm these sample findings. In addition, by only looking at the abstracts we were unable to evaluate whether or not the study’s methodology caused any pain or discomfort to the animals used. Should future research be performed that looks at the entire article, the inclusion of such criteria would make an interesting addition. One other limitation was the inability to view more than 1,000 abstracts from Science Direct for the five searches that returned more than 1,000 results. As these words were still the top five keywords it appears that this limitation had little impact, other than potentially affecting the order and number of returned results for these keywords. 

### 4.8. Emotions Count

Knowledge of whether animals can experience emotions or possess certain traits seen in humans, gives further weight to their value as sentient, emotional beings. We humans continuously seek to compare animals against our own abilities, whether it is by training chimps to use sign-language or making animals do arithmetic. This anthropocentric view is often why we dismiss animal emotions**,** as we do not recognise their emotional experiences or we consider them to significantly differ from ours and be of less importance. The list of 174 keywords used in this review was not meant to represent a catalogue of sentience indicators. It was however, developed to capitalise upon humans’ anthropocentric nature and accommodate the innate tendency of humans to evaluate and measure animals against our human values. Each of the words included in the list has meaning and value either in terms of human sentience and emotions, or in regards to existing work in the animal sentience field. We hope, therefore, that by using these as a benchmark for measuring the prevalence of sentience and related concepts, we have garnered a greater insight into what is considered important by scientists performing animal research. This in turn provides a powerful tool for animal advocates, advisors and animal welfare scientists, helping us to improve the well-being of the animals in our care. 

Animal sentience is often thought to be an abstract concept, something without real definition or tangible indicators. We hope that this review has gone some way towards dispelling some of these misconceptions by approaching the matter in a new way. Animal sentience forms the foundation of animal welfare science and it is why animals need protection. The results clearly show there are fundamental areas which are not yet being considered. Future research must continue to fill these gaps, particularly for those taxa that we use so much yet know so little about. We have shown how little is known about the experience and promotion of positive emotions in animals, and this is an area of utmost importance to the field of animal welfare. By ignoring positive emotions we are ignoring a valuable part of what it means to be alive. With so much to learn about the subjective minds of animals and the challenges this brings, the future of animal sentience science is certainly an exciting one. 

## Appendix

**Table A1:** The keywords used in the study, including details of their source, valence and whether they returned suitable results.

	Keyword	Origin	Valence	Returned results
**A**	Awe	**Plutchik (1981)**	Positive	**No**
	Amazement	**Plutchik (1981), Parrot (2001)**	Positive	**No**
	Admiration	**Plutchik (1981)**	Positive	**No**
	Acceptance	**Plutchik (1981)**	Neutral	**No**
	Apprehension	**Plutchik (1981), Parrot (2001)**	Negative	**Yes**
	Annoyance	**Plutchik (1981), Parrot (2001), HUMAINE (2006)**	Negative	**Yes**
	Anticipation	**Plutchik (1981)**	Neutral	**Yes**
	Aggressiveness	**Plutchik (1981)**	Negative	**Yes**
	Anger	**Plutchik (1981), Parrot (2001), HUMAINE (2006)**	Negative	**Yes**
	Affection	**Parrot (2001)**	Positive	**No**
	Adoration	**Parrot (2001)**	Positive	**No**
	Attraction	**Parrot (2001)**	Positive	**No**
	Arousal	**Parrot (2001)**	Neutral	**Yes**
	Amusement	**Parrot (2001)**	Positive	**No**
	Astonishment	**Parrot (2001)**	Neutral	**No**
	Aggravation	**Parrot (2001)**	Negative	**No**
	Agitation	**Parrot (2001)**	Negative	**Yes**
	Agony	**Parrot (2001)**	Negative	**No**
	Anguish	**Parrot (2001)**	Negative	**No**
	Alienation	**Parrot (2001)**	Negative	**No**
	Alarm	**Parrot (2001)**	Negative	**No**
	Anxiety	**Parrot (2001), HUMAINE (2006)**	Negative	**Yes**
	Altruism	**WSPA**	Positive	**Yes**
	Affective State	**WSPA**	Neutral	**Yes**
				
**B**	Boredom	**Plutchik (1981), HUMAINE (2006)**	Negative	**Yes**
	Bliss	**Parrot (2001)**	Positive	**No**
	Bitterness	**Parrot (2001)**	Negative	**No**
				
**C**	Contempt	**Plutchik (1981), Parrot (2001), HUMAINE (2006)**	Negative	**No**
	Caring	**Parrot (2001)**	Positive	**No**
	Compassion	**Parrot (2001)**	Positive	**No**
	Cheerfulness	**Parrot (2001)**	Positive	**No**
	Contentment	**Parrot (2001)**	Positive	**Yes**
	Conscious	**WSPA**	Neutral	**No**
	Cognitive Ethology	**WSPA**	Neutral	**No**
				
**D**	Disapproval	**Plutchik (1981)**	Negative	**No**
	Distraction	**Plutchik (1981)**	Neutral	**No**
	Disgust	**Plutchik (1981), Parrot (2001), HUMAINE (2006)**	Negative	**Yes**
	Desire	**Parrot (2001)**	Neutral	**No**
	Delight	**Parrot (2001)**	Positive	**No**
	Dislike	**Parrot (2001)**	Negative	**Yes**
	Depression	**Parrot (2001)**	Negative	**Yes**
	Despair	**Parrot (2001), HUMAINE (2006)**	Negative	**No**
	Dismay	**Parrot (2001**	Negative	**Yes**
	Disappointment	**Parrot (2001), HUMAINE (2006)**	Negative	**No**
	Displeasure	**Parrot (2001)**	Negative	**No**
	Defeat	**Parrot (2001)**	Negative	**No**
	Dejection	**Parrot (2001)**	Negative	**No**
	Distress	**Parrot (2001)**	Negative	**Yes**
	Dread	**Parrot (2001)**	Negative	**No**
	Doubt	**HUMAINE (2006)**	Negative	**No**
				
**E**	Ecstasy	**Plutchik (1981), Parrot (2001)**	Positive	**No**
	Enjoyment	**Parrot (2001)**	Positive	**No**
	Elation	**Parrot (2001)**	Positive	**No**
	Euphoria	**Parrot (2001)**	Positive	**No**
	Enthusiasm	**Parrot (2001)**	Positive	**No**
	Excitement	**Parrot (2001)**	Positive	**Yes**
	Exhilaration	**Parrot (2001)**	Positive	**No**
	Eagerness	**Parrot (2001)**	Positive	**Yes**
	Enthrallment	**Parrot (2001)**	Positive	**No**
	Exasperation	**Parrot (2001)**	Negative	**No**
	Envy	**Parrot (2001), HUMAINE (2006)**	Negative	**No**
	Embarrassment	**Parrot (2001), HUMAINE (2006)**	Negative	**No**
	Empathy	**WSPA**	Neutral	**No**
	Emotion	**WSPA**	Neutral	**Yes**
				
**F**	Fear	**Plutchik (1981), Parrot (2001), HUMAINE (2006)**	Negative	**Yes**
	Fondness	**Parrot (2001)**	Positive	
	Frustration	**Parrot (2001), HUMAINE (2006)**	Negative	**Yes**
	Fury	**Parrot (2001**	Negative	**No**
	Ferocity	**Parrot (2001**	Negative	**No**
	Fright	**Parrot (2001**	Negative	**No**
				
**G**	Grief	**Plutchik (1981), Parrot (2001)**	Negative	**No**
	Gaiety	**Parrot (2001)**	Positive	**No**
	Glee	**Parrot (2001)**	Positive	**No**
	Gladness	**Parrot (2001)**	Positive	**No**
	Grouchiness	**Parrot (2001)**	Negative	**No**
	Grumpiness	**Parrot (2001)**	Negative	**No**
	Gloom	**Parrot (2001)**	Negative	**No**
	Glumness	**Parrot (2001)**	Negative	**No**
	Guilt	**Parrot (2001), HUMAINE (2006)**	Negative	**No**
	Generosity	**WSPA**	Positive	**No**
				
**H**	Happiness	**Parrot (2001)**	Positive	**No**
	Hope	**Parrot (2001)**	Positive	**No**
	Hostility	**Parrot (2001)**	Negative	**Yes**
	Hate	**Parrot (2001)**	Negative	**No**
	Hopelessness	**Parrot (2001)**	Negative	**No**
	Homesickness	**Parrot (2001)**	Negative	**No**
	Humiliation	**Parrot (2001)**	Negative	**No**
	Horror	**Parrot (2001)**	Negative	**No**
	Hysteria	**Parrot (2001)**	Negative	**No**
	Helplessness	**HUMAINE (2006)**	Negative	**Yes**
	Hurt	**HUMAINE (2006)**	Negative	**No**
	Infatuation	**Parrot (2001)**	Neutral	**No**
	Irritation	**Parrot (2001), HUMAINE (2006)**	Negative	**No**
	Isolation	**Parrot (2001)**	Negative	**No**
	Insecurity	**Parrot (2001)**	Negative	**No**
	Insult	**Parrot (2001)**	Negative	**No**
	Interest	**Plutchik (1981)**	Neutral	**Yes**
				
**J**	Joy	**Parrot (2001), Plutchik (1981)**	Positive	**Yes**
	Jolliness	**Parrot (2001)**	Positive	**No**
	Joviality	**Parrot (2001)**	Positive	**No**
	Jubilation	**Parrot (2001)**	Positive	**No**
	Jealousy	**Parrot (2001)**	Negative	**No**
				
**K**				
**L**	Love	**Parrot (2001), Plutchik (1981)**	Positive	**No**
	Loathing	**Parrot (2001), Plutchik (1981)**	Negative	**No**
	Liking	**Parrot (2001)**	Positive	**No**
	Lust	**Parrot (2001)**	Positive	**No**
	Longing	**Parrot (2001)**	Negative	**No**
	Loneliness	**Parrot (2001)**	Negative	**No**
				
**M**	Misery	**Parrot (2001)**	Negative	**No**
	Melancholy	**Parrot (2001)**	Negative	**No**
	Mortification	**Parrot (2001)**	Negative	**No**
	Morality	**WSPA**	Neutral	**No**
	Mourn	**WSPA**	Negative	**No**
	Modest	**WSPA**	Neutral	**No**
				
**N**	Neglect	**Parrot (2001)**	Negative	**No**
	Nervousness	**Parrot (2001)**	Negative	**Yes**
				
**O**	Optimism	**Parrot (2001), Plutchik (1981)**	Positive	**Yes**
	Outrage	**Parrot (2001)**	Negative	**No**
				
**P**	Pensiveness	**Plutchik (1981)**	Neutral	**No**
	Passion	**Parrot (2001)**	Positive	**No**
	Pleasure	**Parrot (2001)**	Positive	**Yes**
	Pride	**Parrot (2001)**	Positive	**No**
	Pity	**Parrot (2001)**	Negative	**No**
	Panic	**Parrot (2001)**	Negative	**Yes**
	Powerlessness	**HUMAINE (2006)**	Negative	**No**
	Pessimism	**WSPA**	Negative	**Yes**
	Play	**WSPA**	Positive	**Yes**
	Pain	**WSPA**	Negative	**Yes**
	Personality	**WSPA**	Neutral	**Yes**
				
**Q**				
**R**	Rage	**Parrot (2001), Plutchik (1981)**	Negative	**Yes**
	Remorse	**Parrot (2001), Plutchik (1981)**	Negative	**No**
	Rapture	**Parrot (2001)**	Negative	**No**
	Relief	**Parrot (2001)**	Positive	**No**
	Resentment	**Parrot (2001)**	Negative	**No**
	Revulsion	**Parrot (2001)**	Negative	**No**
	Regret	**Parrot (2001)**	Negative	**No**
	Rejection	**Parrot (2001)**	Negative	**No**
	Revenge	**WSPA**	Negative	**No**
	Rationality	**WSPA**	Neutral	**Yes**
				
**S**	Surprise	**Plutchik (1981)**	Neutral	**Yes**
	Sadness	**Plutchik (1981), Parrot (2001)**	Negative	**No**
	Submission	**Plutchik (1981**	Neutral	**Yes**
	Serenity	**Plutchik (1981**	Positive	**No**
	Sentimentality	**Parrot (2001)**	Neutral	**No**
	Satisfaction	**Parrot (2001)**	Positive	**No**
	Scorn	**Parrot (2001)**	Negative	**No**
	Spite	**Parrot (2001)**	Negative	**No**
	Suffering	**Parrot (2001)**	Negative	**Yes**
	Shame	**Parrot (2001), HUMAINE (2006)**	Negative	**No**
	Sorrow	**Parrot (2001)**	Negative	**No**
	Sympathy	**Parrot (2001)**	Neutral	**No**
	Shock	**Parrot (2001)**	Negative	**No**
	Sentience	**WSPA**	Neutral	**No**
	Self-recognition	**WSPA**	Neutral	**Yes**
	Self-awareness	**WSPA**	Neutral	**No**
	Stress	**WSPA**	Negative	**Yes**
				
**T**	Trust	**Plutchik (1981)**	Positive	**No**
	Terror	**Plutchik (1981), Parrot (2001)**	Negative	**No**
	Tenderness	**Parrot (2001)**	Positive	**No**
	Thrill	**Parrot (2001)**	Positive	**No**
	Triumph	**Parrot (2001)**	Positive	**No**
	Torment	**Parrot (2001)**	Negative	**No**
	Tenseness	**Parrot (2001)**	Negative	**Yes**
	Theory of mind	**WSPA**	Neutral	**Yes**
				
**U**	Unhappiness	**Parrot (2001)**	Negative	**No**
	Uneasiness	**Parrot (2001)**	Negative	**No**
				
**V**	Vigilance	**Plutchik (1981)**	Neutral	**Yes**
	Vengefulness	**Parrot (2001)**	Negative	**No**
	Valence	**WSPA**	Neutral	**No**
				
**W**	Wrath	**Parrot (2001)**	Negative	**No**
	Woe	**Parrot (2001)**	Negative	**No**
	Worry	**Parrot (2001), HUMAINE (2006)**	Negative	**No**
				
**X**				
**Y**				
**Z**	Zeal	**Parrot (2001)**	Positive	**No**
	Zest	**Parrot (2001)**	Negative	**No**
